# Vascular SMC-like CAF-derived THBS1 drives tumor-associated neutrophil recruitment to orchestrate an immunosuppressive microenvironment in gastric cancer

**DOI:** 10.1038/s41419-026-08945-1

**Published:** 2026-06-03

**Authors:** Dandan Li, Zeng Zhou, Yanyan Chen, Pan Huang, Lin Yuan, Qi Wang, Xiangang Zhang, Lantian Zhai, Xinqi Li, Lingyun Xia, Weidong Leng, Shanshan Qin

**Affiliations:** 1 Department of Stomatology, Hubei Provincial Clinical Research Center for Umbilical Cord Blood Hematopoietic Stem Cells, Taihe Hospital, Hubei University of Medicine, Shiyan, China; 2 Hubei Key Laboratory of Embryonic Stem Cell Research, School of Basic Medical Sciences, Hubei University of Medicine, Shiyan, China; 3 Laboratory of Tumor Biology, Academy of Bio-Medicine Research, Hubei University of Medicine, Shiyan, China

**Keywords:** Tumour immunology, Cancer microenvironment

## Abstract

The immunosuppressive tumor microenvironment (TME) remains a primary barrier to effective immunotherapy. Tumor-associated neutrophils (TANs) are key contributors to this immunosuppressive landscape; however, the mechanisms governing their recruitment and infiltration into tumor tissues remain poorly understood. In this study, we unravel a pro-tumorigenic role of thrombospondin-1 (THBS1) in gastric cancer (GC) mediated by TAN recruitment. By integrating single-cell RNA sequencing with immunohistochemical analyses, we demonstrated that THBS1 is predominantly expressed in vascular smooth muscle cell (VSMC)-like cancer-associated fibroblast (CAF), with lower expression observed in malignant epithelial cells. Notably, THBS1-expressing CAFs are significantly expanded in GC tissues. Accordingly, THBS1 is upregulated in GC, and its overexpression is clinically associated with malignant progression and poor prognosis. Genetic depletion of THBS1 in CAFs markedly suppressed GC progression both in vitro and in vivo. Furthermore, deconvolution analysis, patient-derived xenograft (PDX) models, and multiplex immunofluorescence (mIHC) revealed a pronounced spatial co-localization between THBS1-positive CAFs and TANs within GC tissues. TAN infiltration was significantly elevated in GC and positively correlated with THBS1 expression levels and adverse survival outcomes. Critically, neutralizing THBS1 with monoclonal antibodies or specifically knocking down THBS1 in CAFs dramatically reduced TAN infiltration in both PDX and nude mouse xenograft models. Mechanistically, THBS1 knockdown in CAFs impaired the autocrine production and secretion of key neutrophil-attracting chemokines, including CXCL1, CXCL5, and CXCL6. In summary, our findings underscore the significance of SMC-like CAF-derived THBS1 as a critical molecular mediator in the dynamic interplay between CAFs and TANs, ultimately driving GC progression.

**Figure:**
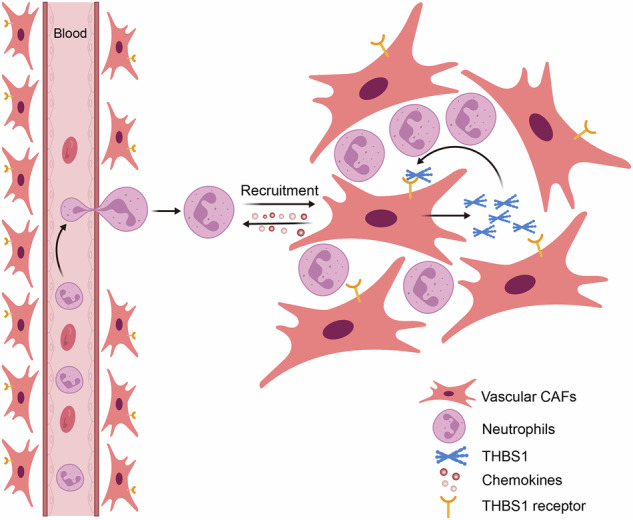

## Highlights

- THBS1 is mainly expressed in VSMCs, CAFs and epithelial cells in GC.
- SNAI2 overexpression results in THBS1 upregulation in GC. The expansion of THBS1-positive CAFs is associated with hyperactivation of the EMT signaling in GC.
- CAFs and TANs exhibit significant spatial co-localization and positively correlated infiltration in GC.
- VSMC-like CAF-derived THBS1 drives GC progression by recruiting TANs.
- Therapeutic blockade of THBS1 via monoclonal antibodies effectively inhibits tumor growth and curtails TAN infiltration, underscoring its potential as a promising therapeutic target in gastric cancer.

## Background

Gastric cancer (GC) is the fifth most common cancer worldwide and the fourth leading cause of cancer-related deaths [1–4]. As a highly heterogeneous disease with significant individual variations, GC is classified into pathological types such as tubular adenocarcinoma, papillary adenocarcinoma, poorly cohesive carcinoma, signet ring cell carcinoma, neuroendocrine carcinoma, and hepatoid stomach cancer [5]. This high heterogeneity encompasses both inter-tumor heterogeneity and intra-tumor cellular heterogeneity, ultimately determining differences in clinical manifestations, treatment responses, and prognoses among patients [6–8]. Therefore, there is an urgent need to develop more effective and precise treatment strategies tailored to the molecular characteristics of different subgroups, aiming to achieve precision medicine.

Neutrophils, derived from the bone marrow stem cells, are polymorphonuclear granulocytes (PMNs). As the “rapid response force” of the human immune system, neutrophils play a critical role in infection and inflammation. Recent studies have revealed that neutrophils exhibit a complex dual role in the tumor immune microenvironment—they can either inhibit tumor growth or promote cancer progression [9]. This functional diversity is closely related to their subtype differentiation, phenotypic plasticity, and modulation by signals from the tumor microenvironment. Within the tumor microenvironment, neutrophils display distinct functional states and are broadly classified into subtypes, including N1 type (anti-tumor function), N2 type tumor-associated neutrophils (TANs, pro-tumor function), and polymorphonuclear myeloid-derived suppressor cells (PMN-MDSCs, pro-tumor function) [10]. TANs can contribute to tumor-promoting inflammation by enhancing angiogenesis, remodeling the extracellular matrix, facilitating metastasis, and establishing an immunosuppressive milieu. Conversely, they may also mediate anti-tumor responses by directly killing tumor cells and participating in cell networks that orchestrate anti-tumor immunity [11].

The thrombospondin (THBS) family consists of five glycoproteins [12], including THBS1-4, THBS5 (COMP). THBS1, a matricellular protein and potent inhibitor of angiogenesis, possesses a multidomain structure that enables interactions with numerous receptors, including integrins, CD36, and CD47 [13]. Dysregulation of THBS1 has been implicated in various diseases, such as cancer, inflammation and metabolic disorders [14]. Nevertheless, the precise role of THBS1 in carcinogenesis remains controversial. Shu and colleagues reported that overexpression of THBS1 inhibits the progression of gallbladder cancer [15]. In contrast, recent studies suggest that THBS1 exerts oncogenic roles in multiple cancers, including breast cancer, glioblastoma, and colon cancer [16–18]. Gong’s group demonstrated that THBS1 facilitates oral squamous cell carcinoma (OSCC) progression by activating the PI3K/Akt signaling pathway [19]. Moreover, high THBS1 expression in mesenchymal cells correlates with poor prognosis and an immunosuppressive microenvironment in colorectal cancer [20]. Conversely, some studies report loss of THBS1 in certain human malignancies, such as renal cell carcinoma [21], and its depletion has been shown to significantly enhance prostate cancer proliferation [22]. Despite these advances, the biological function and specific mechanisms of THBS1 in GC remain poorly understood and warrant further investigation.

It should be noted that THBS1 is a secretory protein. Therefore, it is essential to clarify which cell types express THBS1. Besides, recent studies on THBS1 have highlighted its critical role in the tumor microenvironment. For example, Omatsu et al. reported that tumor-infiltrating monocyte-like cells contribute to immunosuppression and metastasis in colorectal cancer by secreting THBS1 protein [20]. Giraud and colleagues demonstrated that THBS1-expressing myeloid cells contribute to immunosuppression and poor prognosis in hepatocellular carcinoma via TREM1 signaling [23]. Additionally, Liang and Zhang found that THBS1 plays a critical role in CD8 + T cell exhaustion and immune suppression in ovarian cancer [24]. In this study, we uncovered a novel role of vascular smooth muscle cell (VSMC)-derived THBS1 in recruiting tumor-associated neutrophils (TANs). In addition, our findings elucidate the mechanisms underlying THBS1 overexpression in GC and highlight its potential as a promising therapeutic target for GC treatment.

## Methods

### Data acquisition

RNA-seq data and clinical information for the Cancer Genome Atlas (TCGA) stomach adenocarcinoma cohort (STAD) were downloaded from the cBioPortal web server. Transcriptomic data from the GSE62254 dataset were obtained from the Gene Expression Omnibus (GEO) database hosted by NCBI. Clinical information for the ACRG cohort (GSE62254) was extracted from the supplementary materials of Cristescu et al. [25]. Transcriptome data from normal gastric tissues in the GTEx cohort were acquired as previously described [26]. Similarly, transcriptome data from the GSE122401, GSE184336, and GSE179252 cohorts were obtained following our prior protocols [27–29]. For the GSE122401 cohort, normalized gene expression values based on the RSEM (relative standard error of the mean) metric were directly downloaded from the GEO database [30]. For the GSE179252 and GSE184336 cohorts, raw count data were normalized to FPKM (Fragments Per Kilobase of exon model per Million mapped fragments).

### Single-cell RNA sequencing analysis

The single-cell RNA-seq dataset (GSE183904), comprising 31 primary gastric tumor samples spanning clinical stages I–IV and various histological subtypes, was retrieved from the GEO repository [31]. We re-analyzed the raw scRNA-seq data using established pipelines [32]. Briefly, quality control, normalization, and scaling were performed using the Seurat R package (v4.1.0). The 10x Genomics data matrices were imported into Seurat v5.1, followed by cell filtering, sample integration, gene normalization, dimensionality reduction, and visualization. Cells with fewer than 200 or more than 8,000 detected features, or with >20% mitochondrial gene content, were excluded. Principal component analysis (PCA) was conducted using Seurat’s RunPCA function. Clustering and visualization were performed via Uniform Manifold Approximation and Projection (UMAP), using the top 20 principal components (resolution = 0.2 across all merged samples). Cell types were annotated based on canonical markers: CD3D (T cell), TNFRSF17 (Plasma B), EPCAM (Epithelial), MRC1 (Macrophage), PECAM1 (Endothelial), FN1 (Fibroblast), MS4A1 (B cell), and KIT (MAST).

### IHC assay

IHC staining was conducted using Histostain-SP Kits (ZSGB-BIO, China) according to the manufacturer’s protocol. Tissue sections were deparaffinized, rehydrated through graded ethanol, and subjected to antigen retrieval in citrate buffer. Sections were incubated overnight at 4 °C with a primary anti-THBS1 antibody. The next day, HRP-conjugated secondary antibody was applied, and signals were visualized using a DAB kit. Stained slides were imaged using CaseViewer software. Scores for staining intensity: 0 = no staining, 1 = weak staining, 2 = medium staining, 3 = strong staining. Staining intensity was scored as follows: 0 = no staining, 1 = weak, 2 = moderate, 3 = strong. The percentage of positive cells was scored as: 0 = 0–5%, 1 = 6–25%, 2 = 26–50%, 3 = 51–75%, and 4 = 76–100%. The final IHC score was calculated as intensity × percentage. The final IHC score = intensity × percentage.

### Cell culture and transfection

The GC cell lines AGS and HGC-27 were cultured in DMEM (Gibco) supplemented with 10% FBS and 1% penicillin/streptomycin at 37 °C in 5% CO_2_. For overexpression, AGS cells were infected with THBS1-expressing lentivirus (GeneChem) and selected with 2 μg/mL puromycin after 48 h. For knockdown, CAFs were transduced with THBS1-targeting lentivirus (GenePharma) per the manufacturer’s protocol. Cancer-associated fibroblasts (CAFs) were isolated as we previously reported [33]. Fresh GC tissues were washed in PBS containing penicillin (100 U/mL) and streptomycin (100 µg/mL), minced and digested with 0.2% collagenase II/IV and 100 U/mL hyaluronidase at 37 °C for 2 h. Digestion was halted with 10% FBS. After filtration and centrifugation, cells were transferred to a new culture dish. CAFs were resuspended in DMEM/F12 medium (Cat# PM150310, Procell, Wuhan, China) and purified after two passages.

### The cell counting kit-8 assay (CCK-8)

For the Cell Counting Kit-8 assay (CCK-8), GC cell lines were cultured in 96-well plates at 2000 cells per well. At the indicated time points, 10 μL CCK-8 solution was added to each well, and absorbance was measured at 450 nm using a microplate reader. For the wound healing assay, GC cells were seeded into 6-well plates containing complete medium. Once confluence reached 80–90%, a scratch wound was created using a sterile micropipette tip. The wound gap was imaged and quantified at 0, 24, 48 h using a light microscope (Olympus). For the colony formation assay, approximately 500 GC cells per well were seeded in 6-well plates with complete medium. The medium was replaced every three days. After 14 days, the cells were fixed and stained with 0.1% crystal violet for 30 min. The number of cell colonies was then photographed and counted. For the transwell invasion assay, 2 × 10^4^ tumor cells suspended in 200 μL of serum-free medium were seeded into the upper chambers, while the lower chambers were filled with 600 μL complete medium containing 20% FBS. Following incubation, invaded cells on the lower surface were stained with 0.1% crystal violet and imaged and quantified using a light microscope (Olympus).

### RNA extraction and QPCR

Total RNA was extracted using Trizol reagent (TIANGEN, China) and reverse-transcribed into cDNA using Hiscript RT Supermix (Vazyme). qPCR was performed using SYBR Green Master Mix (Vazyme) on a real-time PCR system (Bio-Rad). The relative expression level of target mRNA was calculated using the 2^–ΔΔCt^ method and normalized to β-actin as an internal control. The primer sequences for qPCR analysis are as follows: THBS1 F: TGGTGATGCCTGTGATGATGA; R: TGTTGTCTGTGTCTGCCTGAT; ACTB F: GGATTCCTATGTGGGCGACG; R: GATAGCACAGCCTGGATAGCA.

### Western blotting

Cells were lysed in RIPA buffer (Beyotime) with PMSF. Equal amounts of protein were separated on 8% or 15% SDS-PAGE gels, transferred to PVDF membranes, blocked with 5% milk, and probed overnight at 4 °C with anti-THBS1 (1:1000, #2125, ABclonal). Signals were detected using an ECL kit.

### RNA sequencing

Total RNA from THBS1 knockdown and negative control CAF cells was isolated and analyzed for RNA sequencing. Detailed RNA-seq procedures can be found in the above-mentioned literature [34, 35]. Differential expression analysis (*p* < 0.05 and |log_2_FC | ≥1) was performed using the DESeq2 R package.

### Enzyme-linked immunosorbent assay (ELISA) Assay

CAFs with or without THBS1 knockdown were seeded in a 6-well plate. Upon reaching 80% confluence, the medium was replaced with serum-free DMEM, and the cells were cultured for 24 h to eliminate serum-induced effects on cytokine secretion. Culture supernatants were then collected and transferred to sterile test tubes. Biotinylated detection antibodies were added to form immune complexes with the bound CXCL1/5/6 (RK04787, RK00268, ABclonal, Wuhan; KE00274, Proteintech, Wuhan, China). All experiments were performed in triplicate. Protein concentrations were calculated using a standard curve.

### Chromatin immunoprecipitation (ChIP) assay

ChIP assays were conducted as previously described [36, 37]. Cells were crosslinked with 1% paraformaldehyde for 10 min and quenched with 1×glycine. Cell lysates were then sonicated to shear DNA into fragments. DNA fragments immunoprecipitated using IgG (negative control) or Magnetic beads-conjugated anti-SNAI2 antibody were analyzed by DNA sequencing and qPCR.

### mIHC assay

Multiplex immunohistochemistry (mIHC) assay was conducted using mIF Kits (Servicebio Technology, Wuhan, China). Four-plex immunofluorescence was performed using tyramide signal amplification (TSA) to simultaneously detect four proteins on the same tissue section. The protocol largely followed standard IHC, with TSA labeling and microwave-mediated antibody stripping after each round of primary and secondary antibody incubation. During TSA, HRP-catalyzed oxidation enables fluorescent tyramide to covalently bind near antigen sites; subsequent microwave treatment removes unbound antibodies while preserving the covalently attached fluorophore. This enables sequential, non-cross-reactive labeling with distinct fluorophores. The antibodies and corresponding TSA colors used were: E-cadherin (GB12083, yellow, Servicebio), THBS1 (#2125, green, ABclonal), MMP9 (GB11132-100, cyan, Servicebio), and COL1A1 (GB11022, red, Servicebio).

### Sample collection

This study was approved by the Human Research Ethics Committee of Hubei University of Medicine (Approval No. 2025-PR-061). All patients provided informed consent prior to enrollment. Thirty pairs of GC and adjacent non-tumor tissues were collected from patients who underwent surgical resection at the Department of Gastrointestinal Surgery, Affiliated Taihe Hospital of Hubei University of Medicine. Tissues were immediately snap-frozen in liquid nitrogen and stored at −80 °C until use. All samples were histopathologically confirmed.

### Mouse xenograft model

The animal experiments were approved by the Experimental Animal Research Ethics Committee of Hubei University of Medicine (2025-131). Four-week-old female BALB/c nude mice were purchased from the Laboratory Animal Center of Hubei University of Medicine. For the tumorigenesis assay, 5 × 10^6^ AGS cells with or without THBS1 overexpression were subcutaneously injected into mice. In co-injection experiments, 5 × 10^6^ HGC-27 cells mixed with an equal number of CAFs (with or without THBS1 knockdown) were implanted subcutaneously. After 28 days, mice were euthanized, and tumors were excised for weighing and volume measurement. Tumor volume was calculated as: volume = length × (width)^2^/2.

### Patient-derived xenograft (PDX) model

Fresh tumor tissues from GC patients were collected, cut into small pieces (2 mm^3^), and implanted subcutaneously into nude mice. Once tumors became palpable (typically after one week or longer), mice were randomly divided into two groups. One group received intravenous injections of THBS1 monoclonal antibody every other day for three weeks; the control group received an equivalent amount of isotype control IgG. Subcutaneous tumors were then harvested for mIHC analysis. The use of patient-derived tissues was approved by the Human Research Ethics Committee of Hubei University of Medicine (Approval No. 2025-PR-061).

### Statistical analysis

For gene expression comparisons among GC subtypes, *p* values were calculated using the Mann–Whitney U test. Survival curves were generated by the Kaplan–Meier method and compared using the log-rank test. All other experiments were analyzed by an unpaired *t*-test or a one-way ANOVA test, as appropriate. All experiments were performed with at least three biological replicates and repeated at least three times independently. A *p* value < 0.05 was considered statistically significant.

## Results

### Marked Increase in the proportion of THBS1-high fibroblasts in GC

**Fig. 1:**
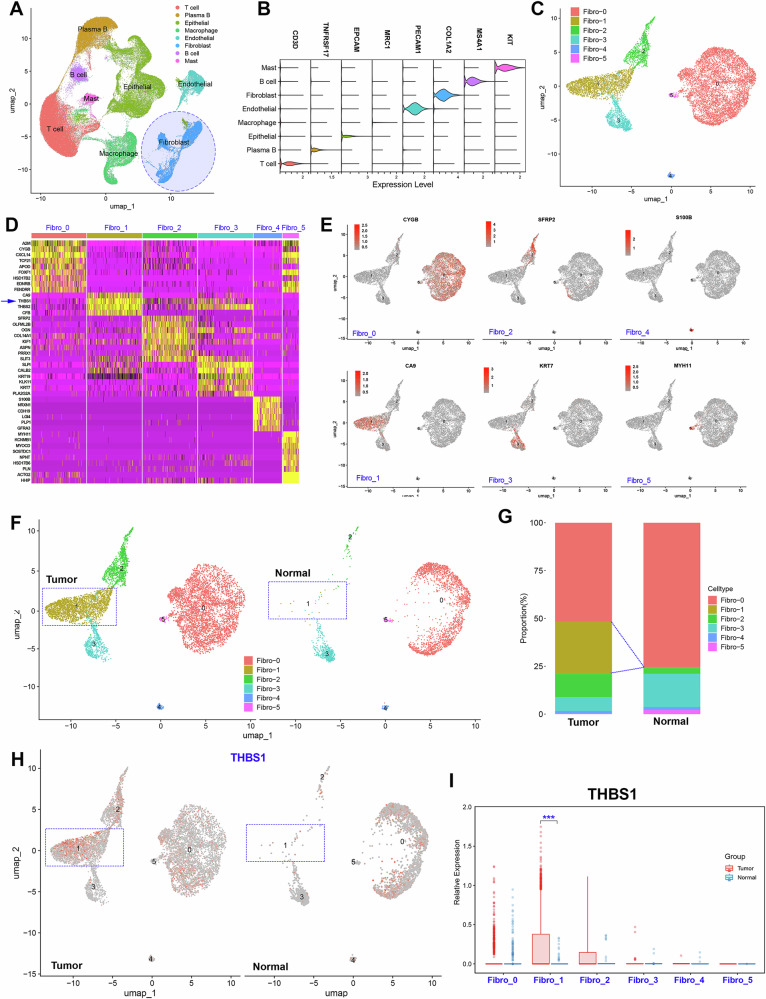
The fibroblast subcluster with high THBS1 expression is significantly expanded in GC. **A** UMAP plots of major cell types from tumor and non-malignant normal tissues of GC patients in the GSE183904 dataset. **B** Expression of canonical marker genes used to annotate major cell lineages. **C** UMAP visualization of 11,353 fibroblasts from the GSE183904 dataset, clustered into six distinct subtypes. **D** Heatmap showing the top differentially upregulated genes in each fibroblast subcluster. **E** UMAP plots depicting the expression of canonical marker genes across fibroblast subclusters. **F** UMAP analysis of fibroblast subclusters derived from tumor versus non-malignant normal samples in GSE183904. **G** Proportional composition of each fibroblast subcluster in tumor and normal tissues. **H** UMAP plot showing THBS1 expression across fibroblast subtypes in GSE183904. **I** THBS1 was significantly upregulated in the Fibro_1 subtype. ***, *P* < 0.001.

Kumar and colleagues have generated a comprehensive single-cell atlas of GC, comprising 31 primary gastric tumor samples [31]. Through reanalysing of the single-cell RNA-seq dataset (GSE183904), we identified eight major cell clusters, including T cell, Plasma B, Epithelial, Macrophage, Endothelial, Fibroblast and Mast cells, with canonical marker gene expression (Fig. 1A, B). The proportion of each cell type within individual samples was presented in Supplementary Fig. S1a. Notably, the proportion of epithelial cells was significantly decreased, whereas that of macrophages was significantly increased in GC (Supplementary Fig. S1B). In contrast, no obvious changes were observed in the proportions of other cell types in GC, including T cells, Plasma B cells, B-cells, and Mast cells, as shown in GC (Supplementary Fig. S1B).

Next, we isolated 11,353 fibroblasts for subclustering analysis. Unsupervised clustering revealed six distinct fibroblast subclusters, designated Fibro_0 through Fibro_5 (Fig. 1C). A heatmap of the top differentially expressed genes in each subcluster is shown in Fig. 1D. Strikingly, the Fibro_1 subcluster exhibited the highest expression levels of both THBS1 and THBS2 (Fig. 1D). UMAP-based heatmaps of key marker genes for each fibroblast subcluster are presented in Fig. 1E. The distribution and abundance of each fibroblast subtype in tumor versus normal tissues are shown in Fig. 1F. Proliferation and proportion analysis further demonstrated that the relative proportions of Fibro_0 and Fibro_3 were significantly decreased in GC, whereas those of the Fibro_1 and Fibro_2 were significantly elevated (Fig. 1G). Notably, the number of Fibro_1 cells, with high THBS1 expression, showed a significant surge in GC (Fig. 1H, I).

### Expression characteristics of THBS1 in GC tissues

**Fig. 2:**
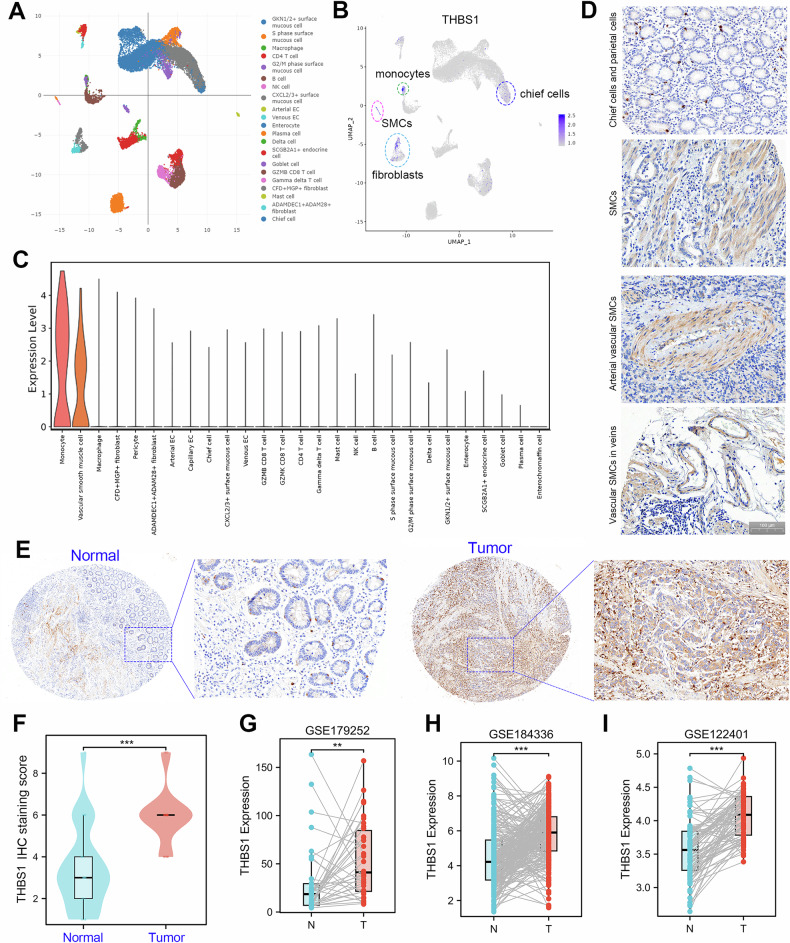
Expression characteristics of THBS1 in GC based on the single-cell analysis and IHC assays. **A** UMAP plot of single cells from gastric tissues annotated using the DISCO database. **B, C** THBS1 is mainly expressed in monocytes, smooth muscle cells (SMC), fibroblasts, and gastric epithelial glandular chief cells. **D** IHC results showed that THBS1 was mainly expressed in chief cells, SMC and VSMC cells. **E** Representative IHC staining of THBS1 in normal gastric tissues and GC tissues. THBS1 is mainly expressed in chief cells in normal gastric tissues, but is widely expressed in nearly all epithelial cells in GC. **F** IHC assays confirmed that THBS1 was overexpressed in GC (*n* = 12). **G–I** THBS1 mRNA was overexpressed in the GSE179252 (*n* = 38), GSE184336 (*n* = 230), and GSE122401 (*n* = 80) stomach cancer cohorts. **, *p* < 0.01, ***, *p* < 0.001.

The DISCO database, developed by Chen and colleagues, provides a comprehensive integration of publicly available human scRNA-seq data across multiple tissues [38]. Using the DISCO platform, we retrieved the expression profile of THBS1 in single-cell data from gastric (cancer) tissues (GSE134520, GSE159929, and GSE167297). Analysis revealed that THBS1 is mainly expressed in monocytes, vascular smooth muscle cells (VSMCs), fibroblasts, and chief cells (Fig. 2A). To validate these scRNA-seq findings, we performed immunohistochemistry (IHC) for THBS1 protein in our GC cohort. As shown in Fig. 2D, THBS1 protein was detected in chief cells, smooth muscle cells (SMCs), and VSMCs of arterial and veins. Consistently, IHC data from the Human Protein Atlas (HPA) database also confirmed THBS1 expression in VSMCs, SMCs, and fibroblasts (Supplementary Fig. S2a).

The expression pattern of THBS1 protein in normal gastric and GC tissues is illustrated in Fig. 2E. In normal tissues, THBS1 was primarily localized to fibroblasts and chief cells within gastric epithelial glands. In contrast, in GC tissues, THBS1 was significantly upregulated in both epithelial cells and fibroblasts (Fig. 2E, F). To further characterize THBS1 mRNA expression in GC, we analyzed three independent cohorts containing paired tumor and adjacent non-tumor samples, including GSE179252 (*n* = 38), GSE184336 (*n* = 230), and GSE122401 (*n* = 80). Consistent with our single-cell and protein-level observations, *THBS1* mRNA was significantly elevated in gastric tumor tissues across all three cohorts (Fig. 2G–I; Supplementary Fig. S2b).

### THBS1 is transcriptionally activated by SNAI2 in GC

**Fig. 3:**
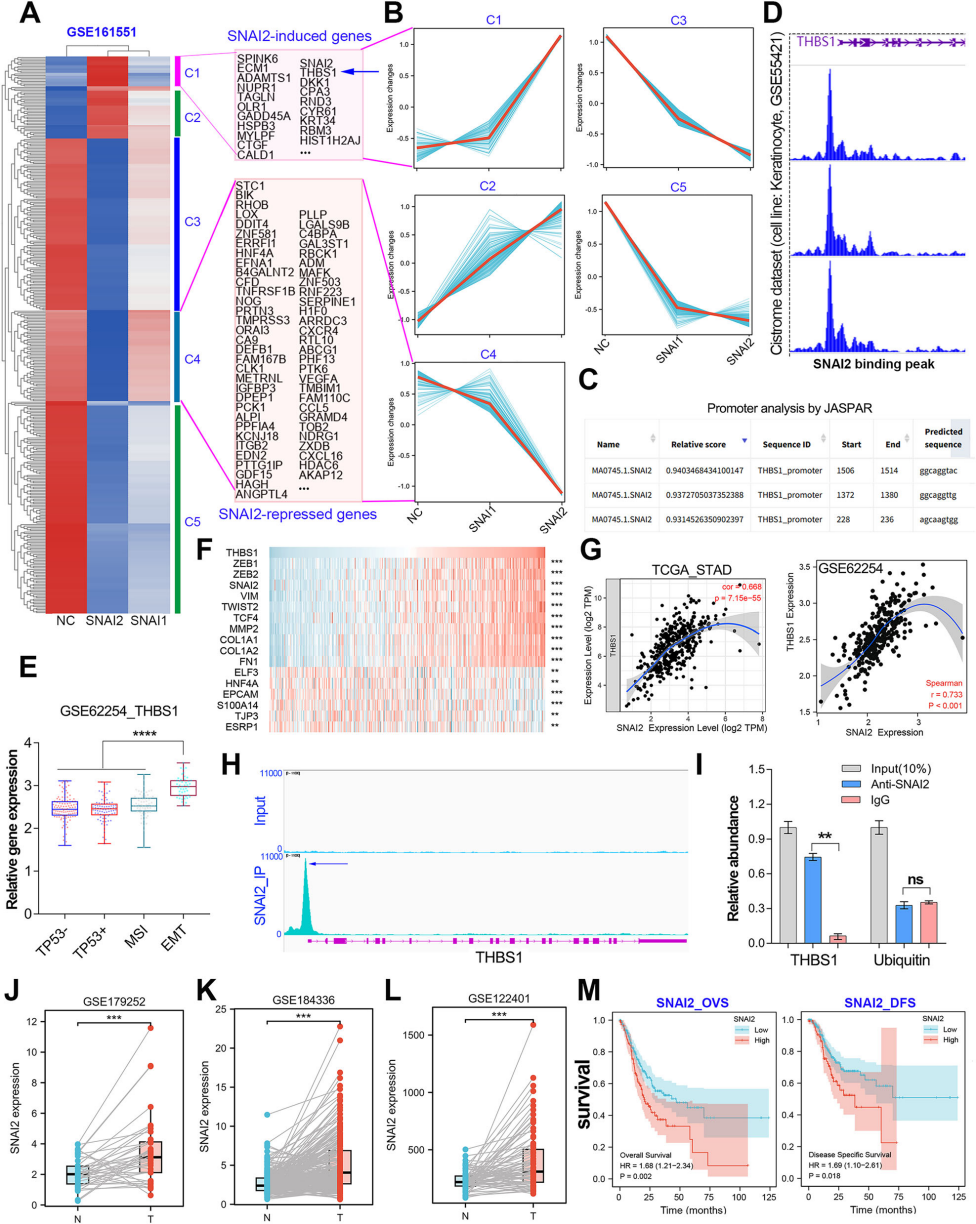
THBS1 expression was transcriptionally activated by SNAI2. **A** Genes regulated by SNAI1/2 (log2FC > 1) were identified based on RNA-seq data (GSE161551) from GC cells with or without SNAI1/2 overexpression. **B** Genes regulated by SNAI1 or SNAI2 were clustered into five subclusters. SNAI2 has a stronger transcriptional regulatory effect than SNAI1 on genes in clusters C3-C5. Cluster C1 and C4 represent genes significantly regulated by SNAI2 alone. **C** Promoter analysis revcealed multiple SNAI2 binding motifs in the *THBS1* promoter. **D** Chip-seq analysis of SNAI2 using the Cistrome web tool showed significant binding of SNAI2 to the *THBS1* promoter. **E** The EMT subtype of GC in the GSE62254 cohort exhibited the highest *THBS1* expression. **F** *THBS1* expression was positively correlated with EMT signaling in the TCGA_STAD cohort. **G** THBS1 was highly co-expressed with SNAI2 in both the TCGA_STAD and GSE62254 cohorts. **H, I** CHIP-seq and qPCR analyses showed that SNAI2 directly binds to the *THBS1* promoter in GC cells. **J–L** SNAI2 mRNA was overexpressed in the GSE179252 (*n* = 38), GSE184336 (*n* = 230), and GSE122401 (*n* = 80) stomach cancer cohorts. **M** SNAI2 overexpression predicted poor prognosis in the TCGA_STAD cohort. ***, *p* < 0.001, ****, *p* < 0.0001.

We previously reported that lncRNA ELF3-AS1 was negatively repressed by transcription factors SNAI1 and SNAI2 in GC, as revealed by RNA-seq analysis [39]. Here, by reanalyzing transcriptomic data from SNAI1- and SNAI2-overexpressing GC cell lines (GSE161551), we comprehensively identified SNAI1/2-regulated target genes and classified them into five distinct subclusters (Fig. 3A, B). C1 represents genes exclusively induced by SNAI2 (including *THBS1*); C2 represents genes activated by both SNAI1 and SNAI2, with stronger induction by SNAI2; C3 and C5 represent genes repressed by both SNAI1 and SNAI2; C4 represents genes exclusively repressed by SNAI2.

Based on these findings, we hypothesized that THBS1 is a direct transcriptional target of the transcription factor SNAI2 in GC. Promoter analysis using the JASPAR webtool predicted three putative SNAI2-binding motifs within the *THBS1* promoter region (Fig. 3C). Consistently, ChIP-seq data from the Cistrome DB revealed a significant SNAI2 binding peak at the THBS1 promoter (Fig. 3D). Cristescu et al. previously classified GC into four molecular subtypes: MSS/TP5 − , MSS/TP53 + , MSI and MSS/EMT subtypes [25]. Analysis of the GSE62254 cohort showed that *THBS1* expression was markedly elevated in the EMT subtype (Fig. 3E). Furthermore, correlation analysis in the TCGA-STAD cohort demonstrated that *THBS1* was positively associated with mesenchymal markers but negatively correlated with epithelial markers (Fig. 3F). Obviously, there was a significant positive co-expression between SNAI2 and THBS1 in both GSE62254 and TCGA_STAD cohorts (Fig. 3G). More importantly, we conducted CHIP-seq analysis in HGC-27 cells and verified a direct physical interaction between SNAI2 and THBS1 promoter (Fig. 3H, I). Taken together, *THBS1* is transcriptionally activated by SNAI2 in GC.

Given that *THBS1* is a downstream target of SNAI2 and is significantly upregulated in GC, we hypothesized that SNAI2 overexpression drives aberrant *THBS1* expression. Indeed, SNAI2 was significantly upregulated in three independent GC cohorts (Fig. 3J–L). Besides, TCGA_STAD patients with high SNAI2 expression possessed a shorter overall survival and disease-free survival time (Fig. 3M). This evidence suggests that the overexpression of SNAI2 contributes to the pathological upregulation of THBS1 in GC.

### THBS1 overexpression predicts poor prognosis in GC

**Fig. 4:**
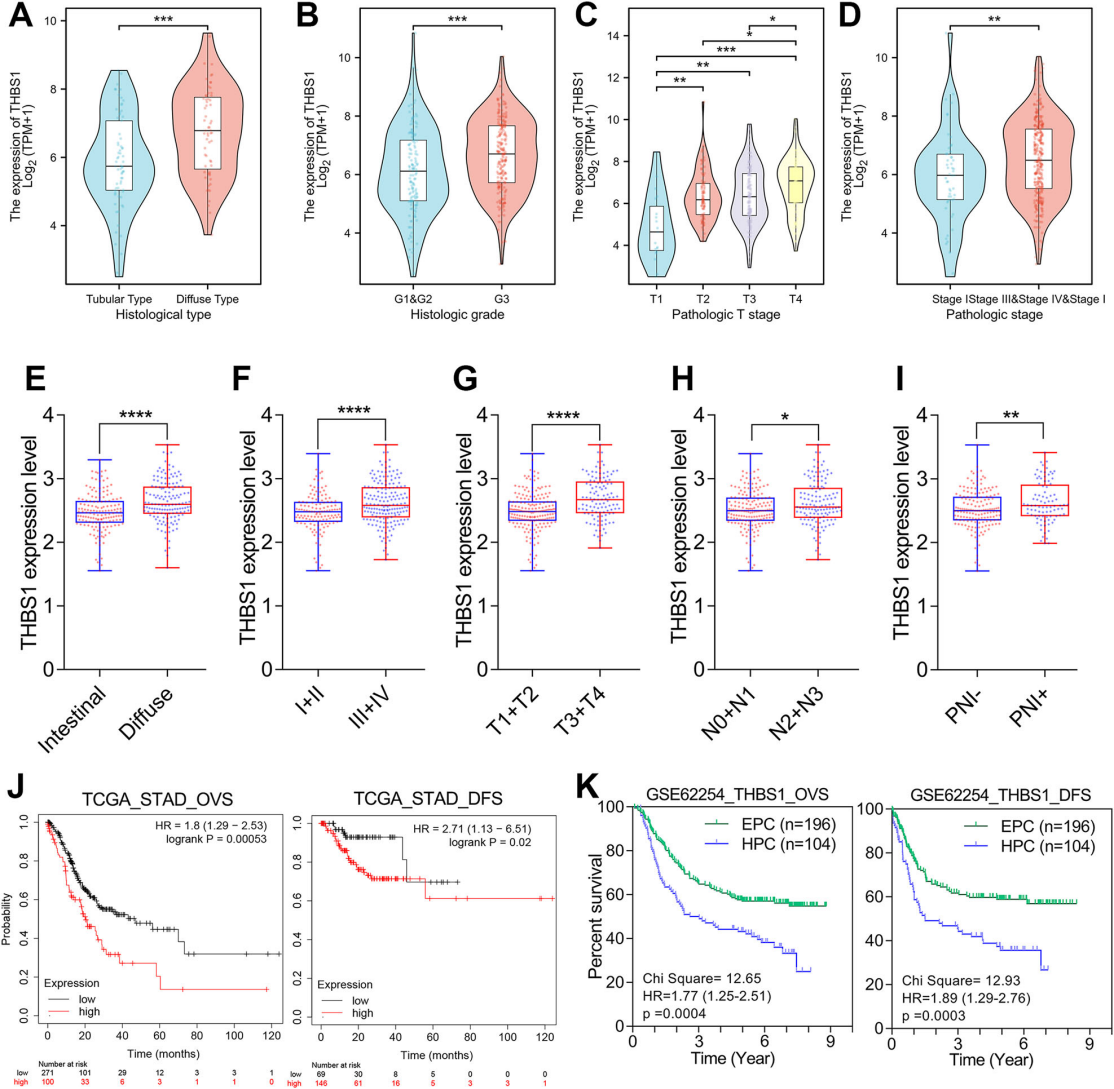
THBS1 overexpression predicted poor survival in GC. **A** THBS1 expression was higher in diffuse-type GC patients than in intestinal GC patients in the TCGA_STAD cohort. **B** The expression of THBS1 is positively correlated with poor differentiation in the TCGA_STAD cohort. **C, D** THBS1 expression is highly expressed in advanced-stage GC and lowly expressed in early-stage GC in the TCGA_STAD cohort. **E** In the GSE62254 cohort, THBS1 was highly expressed in diffuse GC. **F–I** The expression level of THBS1 was positively correlated with pathological stage, T stage, lymph node metastasis, and perineural invasion in the GSE62254 GC patients. **J** THBS1 overexpression predicted poor overall survival (OVS) and disease-free survival (DFS) in the TCGA_STAD cohort. **K** GC patients with high THBS1 expression had shorter OVS and DFS in the GSE62254 cohort. *, *p* < 0.05, **, *p* < 0.01. ***, *p* < 0.001, ****, *p* < 0.0001.

We further analyzed the correlation between THBS1 expression and clinical characteristics as well as prognosis in GC. In the TCGA_STAD cohort, THBS1 was significantly more highly expressed in diffuse-type GC than in intestinal-type GC (Fig. 4A). Patients with poorly differentiated tumors exhibited higher THBS1 expression levels (Fig. 4B). Furthermore, THBS1 expression was significantly elevated in patients with advanced-stage disease compared to those with early-stage GC (Fig. 4C, D).

Consistently, in the GSE62254 cohort, THBS1 was also upregulated in diffuse-type GC (Fig. 4E) and showed a positive correlation with pathological stage, T stage, and lymph node metastasis (Fig. 4F–H). Additionally, GC patients with positive perineural invasion (PNI) displayed significantly higher THBS1 expression (Fig. 4I). As expected, survival analysis across both the TCGA-STAD and GSE62254 cohorts demonstrated that elevated *THBS1* expression was associated with poorer overall survival (Fig. 4J, K). Collectively, these findings suggest that *THBS1* functions as an oncogene in GC.

### THBS1 overexpression Is Associated with an immunosuppressive tumor microenvironment in GC

**Fig. 5:**
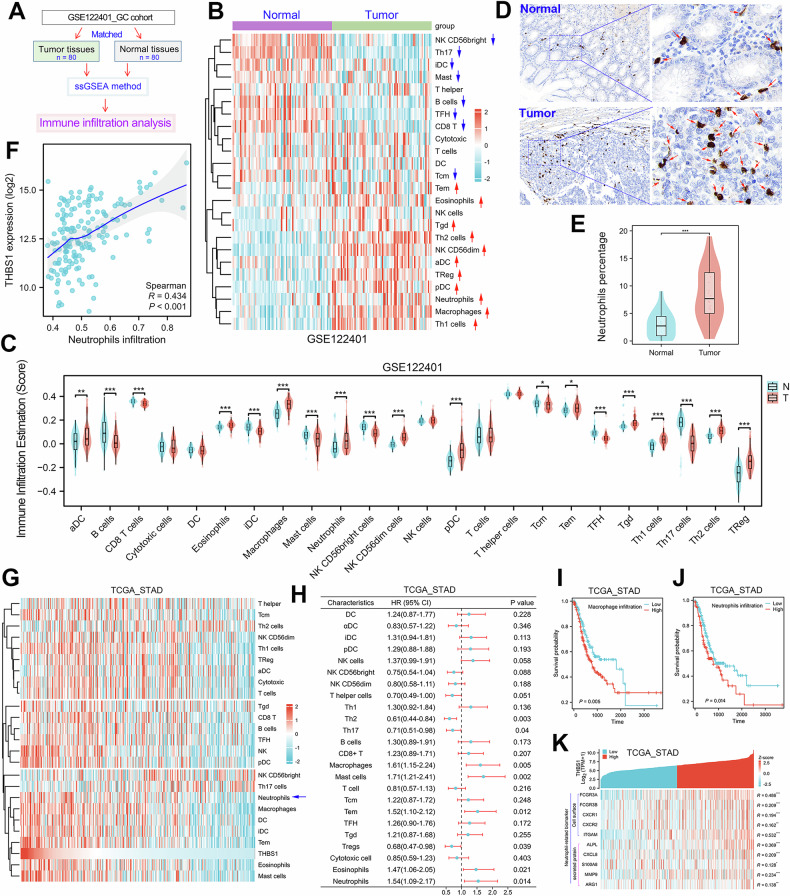
Increased neutrophil infiltration is positively correlated with poor prognosis and THBS1 expression in GC. **A** To evaluate changes in the immune microenvironment during GC progression, we performed deconvolution analysis using the ssGSEA algorithm on transcriptomic data from the GSE122401 cohort, which included tumor and paired adjacent tissues from 80 patients. **B** Heatmap showing infiltration scores of 24 immune cell types in GC and adjacent normal tissues. **C** A total of 19 immune cell types, including neutrophils, showed significant changes in infiltration during GC progression. **D** MMP9 is a marker gene for neutrophils. IHC staining of GC and adjacent normal tissues using an anti-MMP9 antibody revealed neutrophil. The blue arrows represent neutrophils. **E** Neutrophils infiltration was significantly increased in GC. **F** THBS1 expression (log_2_) was positively correlated with neutrophils infiltration in the GSE122401 cohort. **G** Deconvolution analysis using the ssGSEA algorithm on the TCGA_STAD transcriptome revealed a significant positive correlation between THBS1 expression and neutrophil infiltration. **H–J** Prognostic analysis of 24 immune cell types showed that GC patients with high infiltration of macrophages and neutrophils had poor prognosis in the TCGA_STAD cohort. **K** In the TCGA_SATD cohort, the expression level of THBS1 was significantly co-expressed with neutrophil surface marker genes and secreted protein genes. **, *p* < 0.01. ***, *p* < 0.001.

To explore the role of THBS1 in the tumor immune microenvironment (TIME), we performed ssGSEA-based deconvolution analysis on the GSE122401 cohort (Fig. 5A). This method estimates the infiltration levels of 24 distinct immune cell types from bulk transcriptomic data. The immune infiltration landscape across GC and adjacent normal tissues is visualized in the heatmap (Fig. 5B). Notably, the infiltration levels of tumor-associated macrophages (TAMs) and tumor-associated neutrophils (TANs) were significantly increased in tumor tissues (Fig. 5C). Since MMP9 is a well-established biomarker for TANs [40], we validated this finding by immunohistochemistry (IHC). Consistent with the transcriptomic data, IHC staining for MMP9 confirmed a marked increase in TAN infiltration in GC (Fig. 5D, E; Supplementary Fig. S3).

The immunosuppressive TIME is characterized by exhaustion of effector immune cells and enrichment of immunosuppressive populations, among which TAMs and TANs are key drivers of immune evasion [41–44]. Interestingly, THBS1 expression was significantly positively correlated with the infiltration levels of both TAMs (Supplementary Fig. S4) and TANs in the GSE122401 cohort (Fig. 5F). A similar positive correlation was observed in the TCGA-STAD cohort (Fig. 5G). Consistently, prognostic analysis revealed that GC patients with high TAMs or TANs infiltration had significantly worse clinical outcomes (Fig. 5H–J). Furthermore, *THBS1* expression showed strong positive correlations with surface markers and secreted proteins characteristic of TANs (Fig. 5K). These results suggest that high *THBS1* expression is closely linked to an immunosuppressive tumor microenvironment in GC.

### THBS1 depletion in CAFs impairs neutrophil recruitment and tumor formation

**Fig. 6:**
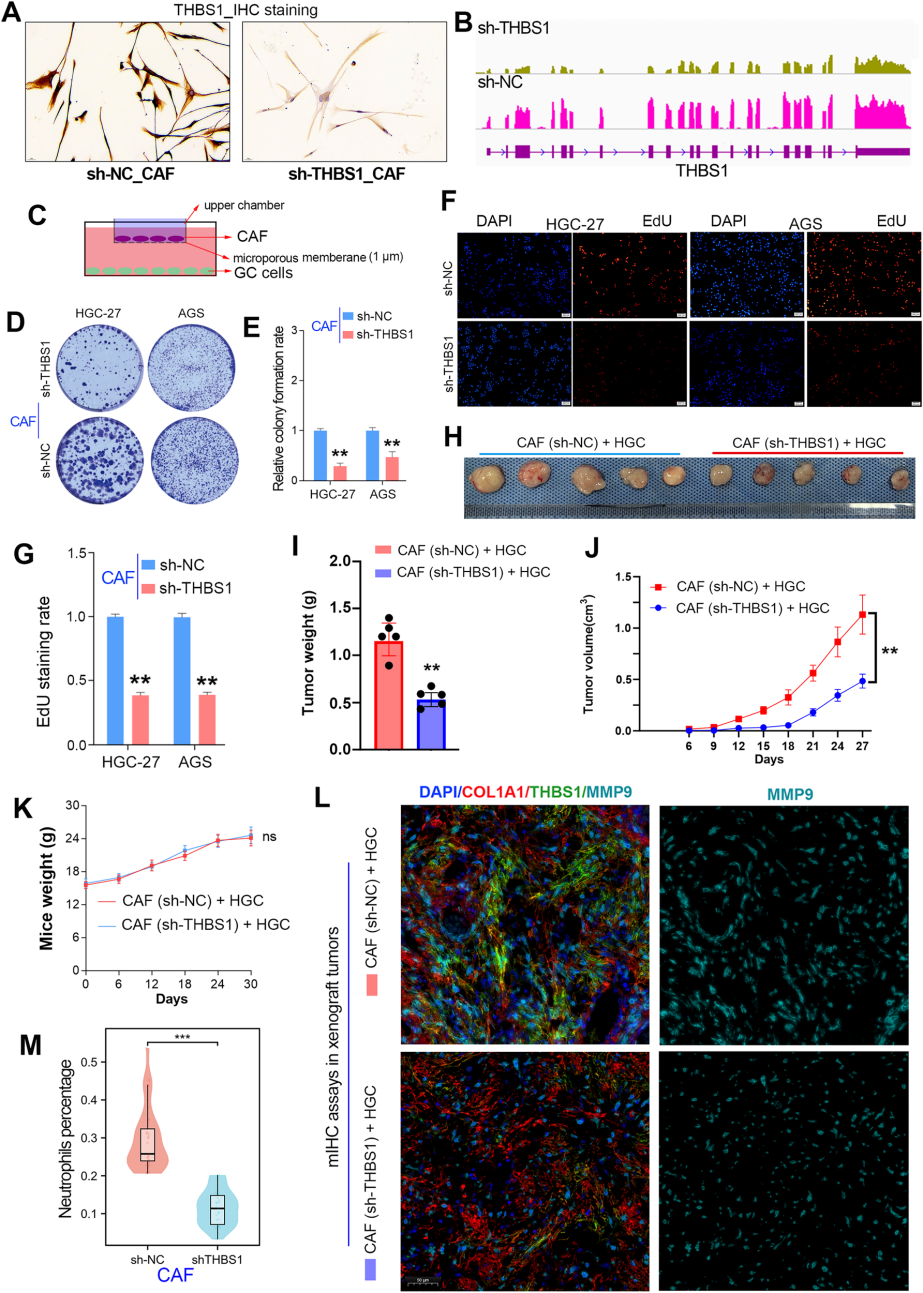
Knockdown of THBS1 in CAFs significantly inhibits GC proliferation and neutrophil infiltration. **A, B** IHC and RNA-seq analysis confirmed successful THBS1 knockdown in CAFs. **C** Schematic diagram of co-culture system for CAFs and GC Cells. **D–G** Knockdown of THBS1 in CAFs significantly attenuated their ability to promote GC cell proliferation in vitro. **H–J** In a xenograft tumor model, CAF cells and GC cells were co-inoculated subcutaneously into nude mice at a 1:1 ratio. CAFs with THBS1 knockdown significantly suppressed xenograft tumor size, weight, and volume. **K** No significant difference in body weight was observed between the control and the THBS1-knockdown groups. **L, M** Knockdown of THBS1 in CAFs significantly reduced neutrophil infiltration in xenograft tumors. **, *p* < 0.01, ***, *p* < 0.001.

As shown in the right panel of Fig. 2A–E, THBS1 is expressed in both fibroblasts and epithelial cells. To investigate the functional role of fibroblast-derived THBS1 on tumor growth and TANs infiltration, we isolated primary cancer-associated fibroblasts (CAFs) from fresh GC specimens and generated stable THBS1-knockdown CAF lines using lentiviral transduction. The IHC assays confirmed efficient knockdown of THBS1 protein in CAFs (Fig. 6A), and RNA-seq analysis further validated a significant reduction of *THBS1* mRNA levels in CAFs (Fig. 6B).

Then, the HGC-27 and AGS GC cell lines were co-cultured with either control or THBS1-knockdown CAFs (Fig. 6C). The colony formation and EdU staining assays together showed that knockdown of THBS1 significantly inhibited the promoting effect of CAF cells on GC cell proliferation in vitro (Fig. 6D–G). To assess the impact of CAF-derived THBS1 on tumorigenesis in vivo, we co-injected HGC-27 cells and CAFs (1:1 ratio) subcutaneously into nude mice. One month after inoculation, tumors derived from THBS1-knockdown CAFs exhibited significantly reduced growth compared to those from control CAFs, indicating impaired tumor-promoting capacity (Fig. 6H–J). Notably, THBS1 knockdown in CAFs did not affect the body weight of nude mice, suggesting no systemic toxicity (Fig. 6K).

Next, we performed multiplex immunofluorescence (mIHC) analysis on xenograft tumors from the THBS1 knockdown group and the control group. Specifically, blue light (DAPI) was used to label cell nuclei; red light (COL1A1) for labeling fibroblasts; green light (THBS1) to detect THBS1 expression levels in CAF cells; and cyan light (MMP9) for labeling TANs (Fig. 6L). Notably, compared with the controls, THBS1-knockdown xenografts showed markedly reduced green fluorescence (indicating lower THBS1 expression) and a significant decrease in MMP9⁺ TAN infiltration (Fig. 6L, M). These in vitro and in vivo evidence together confirmed that CAF-derived THBS1 plays an essential role in promoting gastric tumor growth and recruiting TANs.

### THBS1 overexpression in cancer cells promotes GC progression

**Fig. 7:**
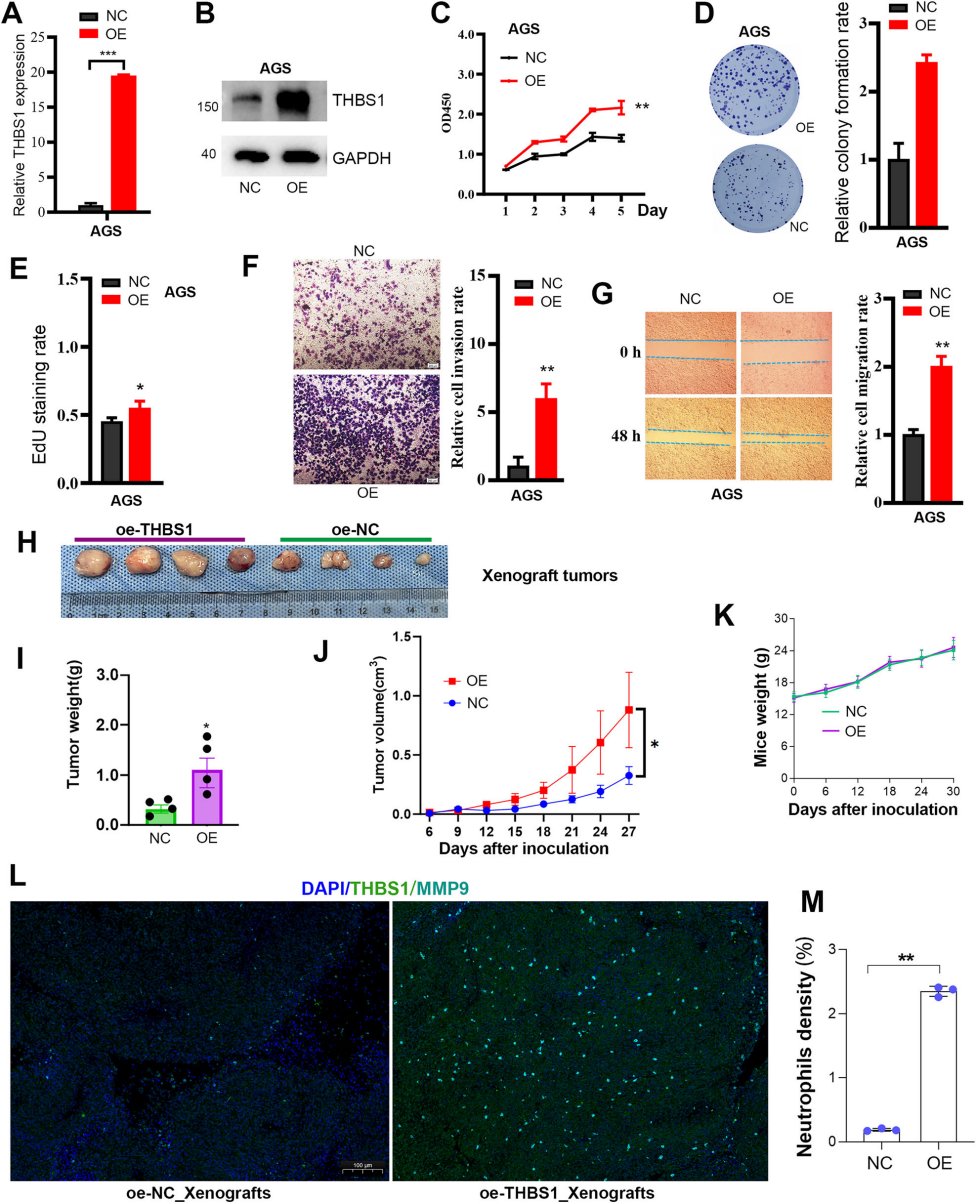
THBS1 overexpression in cancer cells promotes GC progression in vitro and in vivo. **A, B** qPCR and western blotting assays confirmed successful THBS1 overexpression in the AGS GC cell line. **C–E** CCK-8, colony formation, and EdU staining assays showed that THBS1 overexpression promotes AGS cell proliferation. **F, G** Transwell invasion and wound healing assays demonstrated that THBS1 overexpression enhanced AGS cell invasion and migration. **H–J** Equal amounts of control and THBS1-overexpressing AGS cells were subcutaneously inoculated into nude mice. THBS1 overexpression significantly increased xenograft tumor size, weight, and volume. **K** No significant difference in body weight was observed between the control and the THBS1 overexpression groups. **L, M** Neutrophil infiltration was significantly higher in xenograft tumors from the THBS1-overexpression group than in the control group. **, *p* < 0.01.

As noted earlier, THBS1 is also overexpressed in the GC epithelial cells. However, whether tumor cell–derived THBS1 contributes to TAN recruitment remains unclear. To address this, we established a GC epithelial cell line (AGS) with stable THBS1 overexpression (Fig. 7A, B). The CCK-8, colony formation, and EdU assays confirmed that THBS1 overexpression significantly promoted GC cell proliferation (Fig. 7C–E). Transwell and wound-healing assays indicated that THBS1 overexpression enhanced the invasive and migratory abilities of GC cells (Fig. 7F, G). In parallel, we performed subcutaneous xenograft experiments in nude mice using THBS1-overexpressing or control AGS cells. In vivo results demonstrated that THBS1 overexpression significantly accelerated xenograft tumor growth without affecting the body weight of nude mice (Fig. 7H–K). As expected, mIHC assays showed that the THBS1-overexpression group exhibited stronger green signal and a concomitant increase in MMP9⁺ TAN infiltration compared to controls (Fig. 7L, M). Notably, the proportion of neutrophils in xenograft tumors derived solely from GC cells (1-3%) was substantially lower than that in tumors formed by CAF&HGC-27 co-culturing cells (20-50%).

### CAF recruits neutrophils by secreting THBS1 in an autocrine manner

**Fig. 8:**
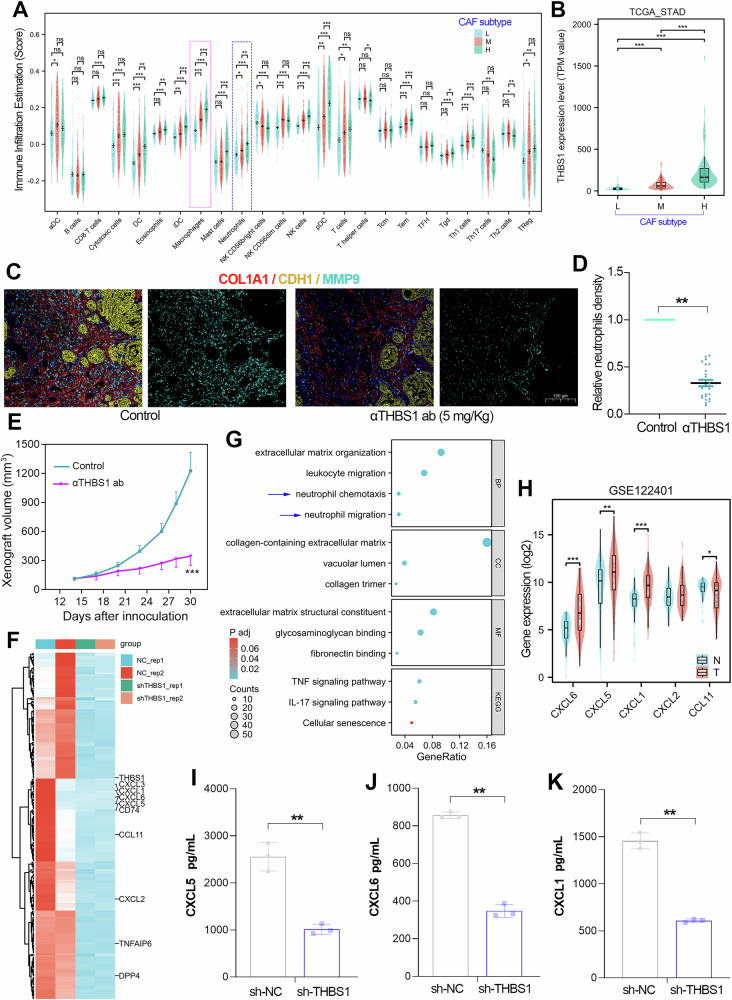
THBS1 recruits neutrophils by enhancing chemokine secretion in an autocrine manner. **A, B** In the TCGA_STAD cohort, GC subtypes with high CAF infiltration showed elevated neutrophil infiltration and THBS1 expression. **C** We established a GC patient-derived PDX model in nude mice and randomly assigned mice to receive either THBS1 monoclonal antibody or control treatment. mIHC was performed on PDX xenografts to assess the effect of THBS1 blockade on neutrophil infiltration. mIHC results revealed significant co-localization of neutrophils and fibroblasts. **D** Neutrophil infiltration was significantly reduced in xenografts from the THBS1 monoclonal antibody–treated group. **E** Tumor volume measurements in the PDX model comparing the THBS1 monoclonal antibody–treated and control groups. **F** RNA sequencing of THBS1-knockdown and control CAF cells revealed differentially expressed genes, with multiple chemokines highlighted in the heatmap. **G** Genes significantly downregulated upon THBS1 knockdown were enriched in pathways related to neutrophil chemotaxis and migration. **H** Gene expression analysis showed that among the chemokines regulated by THBS1, the expression levels of CXCL1, CXCL5, and CXCL6 were significantly upregulated in GC. **I–K** ELISA assays on CAF culture supernatants confirmed elevated secretion of CXCL1, CXCL5, and CXCL6. **, *p* < 0.01, ***, *p* < 0.001.

We previously reported a molecular classification method for CAF heterogeneity in GC [26]. According to the degree of CAF infiltration, GC patients can be divided into high-, medium-, and low-CAF infiltration groups. Here, we comprehensively compared the TIME landscapes across these three groups. As expected, the high-CAF infiltration group exhibited the highest levels of TAN and TAM infiltration, whereas the low-CAF infiltration group showed the lowest levels (Fig. 8A). Similarly, THBS1 expression was highest in the high-CAF infiltration group and lowest in the low-CAF infiltration group (Fig. 8B).

In our co-culture system (Fig. 6C–G), a microporous membrane with 1-μm pores separates the upper and lower chambers, allowing secreted proteins to diffuse freely between compartments. This led us to hypothesize that THBS1 knockdown in CAFs might alter their secretomes, thereby inhibiting GC cell proliferation and metastasis. To test this, we performed patient-derived xenograft (PDX) experiments by directly transplanting fresh GC tumor tissues into nude mice. Starting on day 7 post-inoculation, mice received intraperitoneal injections of a THBS1 monoclonal antibody (5 mg/kg) every other day to block THBS1 signaling. Three weeks later, we excised the xenograft tumors and performed mIHC analysis. Among them, blue light (DAPI) was used to label cell nuclei; red light (COL1A1) for labeling fibroblasts; yellow light (CDH1) for labeling epithelial-derived tumor cells; and cyan light (MMP9) for labeling TANs. Notably, THBS1 blockade significantly reduced neutrophil infiltration compared to the control group (Fig. 8C, D). Importantly, the spatial distribution of TANs closely overlapped with that of fibroblasts (Fig. 8C), suggesting that CAFs actively recruit neutrophils. In addition, THBS1 monoclonal antibody treatment markedly suppressed xenograft tumor growth in the PDX model (Fig. 8E).

To elucidate the underlying role of CAF-derived THBS1 in promoting TAN recruitment, we performed RNA-seq studies on control and THBS1-knockdown CAFs. The heatmap revealed significant downregulation of multiple chemokine genes (CXCL1, CXCL2, CXCL3, CXCL5, CXCL6, CCL11) following THBS1 knockdown (Fig. 8F). GO/KEGG analysis further indicated that the differentially expressed genes in CAFs after THBS1 depletion were significantly enriched in pathways related to neutrophil chemotaxis and migration (Fig. 8G). Among these THBS1-regulated chemokines, *CXCL6*, *CXCL5*, and *CXCL1* were notably upregulated in GC tissues (Fig. 8H). ELISA measurements of CAF-conditioned media confirmed that secretion of CXCL6, CXCL5, and CXCL1 was significantly reduced upon THBS1 knockdown (Fig. 8I–K). Given the well-established roles of CXCL6, CXCL5, and CXCL1 in TAN recruitment, we conclude that CAF-derived THBS1 acts in an autocrine manner to promote the secretion of these chemokines, thereby recruiting TANs, fostering an immunosuppressive tumor microenvironment, and driving GC progression.

## Discussion

The tumor immune microenvironment (TIME) refers to a complex ecosystem composed of diverse cell types, signaling molecules, and extracellular matrix that surrounds tumor cells [33]. The efficacy of traditional anti-cancer therapy approaches in GC is often limited and frequently accompanied by severe side effects, making immunotherapies—capable of specifically targeting tumor cells—highly promising [27, 45, 46]. Nevertheless, due to the high heterogeneity of GC, the current progress in immunotherapy has remained suboptimal over the past decade [47, 48]. The therapeutic efficacy of immunotherapy in GC critically depends on the composition and functional state of the TIME [49]. Beyond providing structural support for tumor growth, the TIME dynamically regulates immune responses and thereby influences tumor initiation, progression, and treatment outcomes [50]. Consequently, remodeling the TIME can convert immunologically “cold” tumors—insensitive to immunotherapy—into “hot” tumors that are responsive to immune checkpoint blockade, ultimately improving patient prognosis [51–53].

The primary obstacle to immunotherapy is the immunosuppressive tumor microenvironment [51]. Activated neutrophils, including polymorphonuclear myeloid-derived suppressor cells (PMN-MDSCs) and N2 tumor-associated neutrophils (TANs), constitute key cellular components of this immunosuppressive niche [44]. Meng et al. reported that the immunosuppressive CD10^+^ALPL^+^ neutrophils promote immunotherapy resistance in liver cancer by driving T cell exhaustion [43]. In pancreatic ductal cancer, TANs establish an immunosuppressive microenvironment through upregulation of Nectin-2 [54]. Similarly, Wang and colleagues reported that TANs foster immune suppression in GC by the GM-CSF-PDL1 signaling pathway [55]. However, these studies did not reveal the mechanisms underlying TAN recruitment into tumors.

Our previous studies found that GC patients with high CAF infiltration exhibit poorer prognoses [26]. Additionally, CAFs drive malignant progression across multiple tumor types by secreting cytokines, growth factors, and extracellular proteins [45]. These CAF-derived secretomes can also play an essential role in TIME remodeling through multiple mechanisms [56]. Specifically, CAFs facilitate tumor immune evasion by: (i) recruiting immunosuppressive immune cells, (ii) suppressing effector immune cell function, (iii) forming matrix barriers, and (iv) inducing metabolic remodeling [57–59]. Importantly, CAFs are highly heterogeneous, and distinct CAF subsets exhibit unique functional properties [57]. Thus, in-depth characterization of CAF heterogeneity and their interaction networks with immune cells is expected to uncover novel therapeutic targets for overcoming immunotherapy resistance and for developing rational “CAF-immunotherapy combination strategies”.

In this study, through a comprehensive analysis of the high-quality single-cell data (GSE183904) in GC [31], we identified a unique CAF subset characterized by high expression of THBS1 and THBS2, which is significantly expanded in GC. Accumulating evidence suggests that epithelial cells can undergo epithelial–mesenchymal transition (EMT) to transdifferentiate into fibroblast-like cells [60]. Consistently, our recent study reported that EMT signaling is hyperactivated in GC, accompanied by a reduction in epithelial cells [61]. SNAI2 is a well-established master transcription factor that drives EMT signaling during tumorigenesis and metastasis [3]. During the identification of SNAI2 downstream target genes, we found that THBS1—but not THBS2—could be specifically transcriptionally induced by SNAI2 (Fig. 3). The expansion of THBS1-positive CAFs may result from SNAI2-mediated activation of the EMT signaling pathway, which drives the transdifferentiation of epithelial cells into fibroblasts.

In the present study, we focused on the role of vCAF-derived THBS1 in the gastric tumor microenvironment. IHC staining and single-cell analysis confirmed that THBS1 is predominantly expressed in vascular smooth muscle cells (VSMCs) and smooth muscle cells (SMCs). Zhang and colleagues identified six distinct fibroblast subsets, the majority of which were vascular CAFs (vCAFs) exhibiting high expression of MCAM/CD146 [62]. Consistent with this, our reanalysis of the DISCO dataset revealed strong *MCAM* expression in SMCs (Supplementary Fig. S5), suggesting that vCAFs represent a VSMC-like CAF population.

Consistent with prior reports [13, 63–65], our results also demonstrated that THBS1 is overexpressed in GC and positively correlated with lymph node metastasis and poor prognosis. Epithelial-mesenchymal transition (EMT) is recognized as the initial step for tumor cells' metastasis [3, 61, 66]. In the present study, we identified EMT-related transcription factor SNAI2 as an upstream regulator of THBS1 via transcriptional activation. Accordingly, THBS1 expression was positively correlated with the EMT signaling pathway and metastasis progression in GC. Notably, SNAI2 overexpression resulted in significant upregulation of both THBS1 mRNA and protein levels in GC.

Furthermore, our single-cell analysis further revealed a marked reduction in the proportion of epithelial cells in GC tissues (Supplementary Fig. S1a, b), accompanied by a significant expansion of the Fibro-1 subcluster (Fig. 1G). Previous studies have shown that mesenchymal cells derived from epithelial cells via EMT constitute an important source of fibroblasts [67–70]. We therefore speculate that SNAI2-driven activation of the EMT program may contribute to the loss of epithelial identity and the concomitant expansion of Fibro-1 fibroblasts.

Although THBS1 has been implicated in the regulation of TIME, its potential role in neutrophil functional modulation remains unreported. Here, we uncovered a novel function of THBS1 in promoting neutrophils recruitment. Deconvolution analysis and IHC results collectively showed that neutrophil infiltration was significantly increased in GC and associated with poor clinical outcomes. Moreover, THBS1 expression levels exhibited a strong positive correlation with neutrophil abundance. According to the single-cell analysis, THBS1 is primarily expressed in monocytes and vascular smooth muscle cells (VSMCs), with only low-level expression detected in epithelial cells (Fig. 2C). Consistent with this finding, in the in vivo tumorigenesis experiments, VSMC-like CAFs—rich in THBS1—recruited a substantially higher abundance of neutrophils (20–50%) compared to epithelial cells (1–3%). Moreover, in patient-derived xenograft (PDX) models, neutrophils show substantial spatial co-localization with CAFs—rather than with epithelial cells (Fig. 8C). Therefore, THBS1 derived from VSMC-like CAFs primarily functions within the tumor interior by recruiting TANs to establish an immunosuppressive microenvironment, thereby enabling tumor cells to evade CD8⁺ T cell–mediated cytotoxicity. In contrast, tumor cell–derived THBS1 is expressed at relatively low levels and exhibits minimal capacity to recruit TANs. Li et al. reported that knockdown of THBS1 in GC cell lines inhibited cell proliferation and migration by inducing pyroptosis in tumor cells [71]. In this study, cellular model experiments confirmed that THBS1 overexpression still significantly promotes tumor growth and metastasis even in the absence of neutrophils (Fig. 7C-G).

Multiplex IHC analysis of both xenograft and PDX tumors revealed that neutrophils were densely localized in fibroblast-rich regions. This observation is further supported by our CAF molecular subtyping data, which showed that tissues with high CAF infiltration exhibit elevated THBS1 expression and increased neutrophil infiltration. Moreover, knockdown of THBS1 in either CAFs or tumor cells significantly inhibited xenograft tumor growth and reduced neutrophil infiltration. Critically, in PDX models, neutralization of secreted THBS1 using a monoclonal antibody significantly reduced both intratumoral neutrophil levels and PDX tumor size. These results together implied that THBS1 plays a critical role in neutrophils infiltration.

RNA-seq analysis confirmed that THBS1 knockdown leads to decreased expression of multiple chemokines. GO/KEGG analysis revealed that genes downregulated upon THBS1 knockdown were significantly enriched in neutrophil recruitment-related signaling pathways. ELISA assays demonstrated that knockdown of THBS1 significantly suppressed the secretion of CXCL1, CXCL5, and CXCL6—chemokines that are markedly upregulated in GC. Chemokines have been reported to be involved in neutrophil recruitment [11]. Therefore, we propose that THBS1 promotes neutrophil infiltration by enhancing the secretion of these key chemokines.

A major limitation of this study is that it remains unclear how THBS1 precisely regulates the expression and secretion of chemokines. Numerous studies have confirmed that chemokine expression is tightly regulated by inflammation-related signaling pathways, such as NF-κB, MAPK, JAK/STAT, and IRF pathways. According to GO/KEGG enrichment analysis in Fig. 8G, the DEGs following THBS1 knockdown in CAF were significantly enriched in the TNF and IL-17 signaling pathways, both of which are associated with inflammation. Accordingly, numerous studies have underscored the pivotal role of THBS1 in activating inflammatory signaling pathways in diverse diseases [72–75]. Therefore, a possible explanation is that THBS1 regulates chemokine expression by binding to its receptors—such as CD36, CD47, or integrin proteins, thereby activating inflammation signaling pathways. Nevertheless, the detailed molecular mechanisms by which THBS1 regulates chemokines warrant further investigation.

Increasing evidence confirmed that neutrophils exerting tumor-promoting effects in GC through multiple mechanisms. First, neutrophil can drive GC progression by forming neutrophil extracellular traps (NETs) [76–78]. Second, neutrophils can directly interact with tumor cells to activate the EMT signaling pathway [79]. Third, neutrophils facilitate immune evasion by upregulating PD-L1 expression [55]. Additionally, neutrophils also play a role in T helper cells differentiation by highly expressing ICOSLG [80]. Neutrophils promote tumorigenesis in GC through exosomes and secreted factors such as IL-17 [81, 82]. Within TIME, neutrophils can be polarized into anti-tumor (N1 phenotype) or pro-tumor (N2 phenotype) phenotypes. In this study, we demonstrated that THBS1 recruits N2-polarized TANs by enhancing chemokine secretion, thereby promoting GC progression. Taken together, our findings highlight THBS1 as a promising immunotherapeutic target for GC, with therapeutic potential achieved through inhibition of TAN recruitment.

## Supplementary information

Original WB gels

Supplementary Figures S1-S5 and legends

## Data Availability

The datasets generated during the current study are available in this article or in supplementary files.
