# Supplementary material for: Vascular SMC-like CAF-derived THBS1 drives tumor-associated neutrophil recruitment to orchestrate an immunosuppressive microenvironment in gastric cancer
Source: Cell Death Dis. 2026 Jun 3;17(1):694. doi: 10.1038/s41419-026-08945-1 (PMC13448681; doi:10.1038/s41419-026-08945-1)

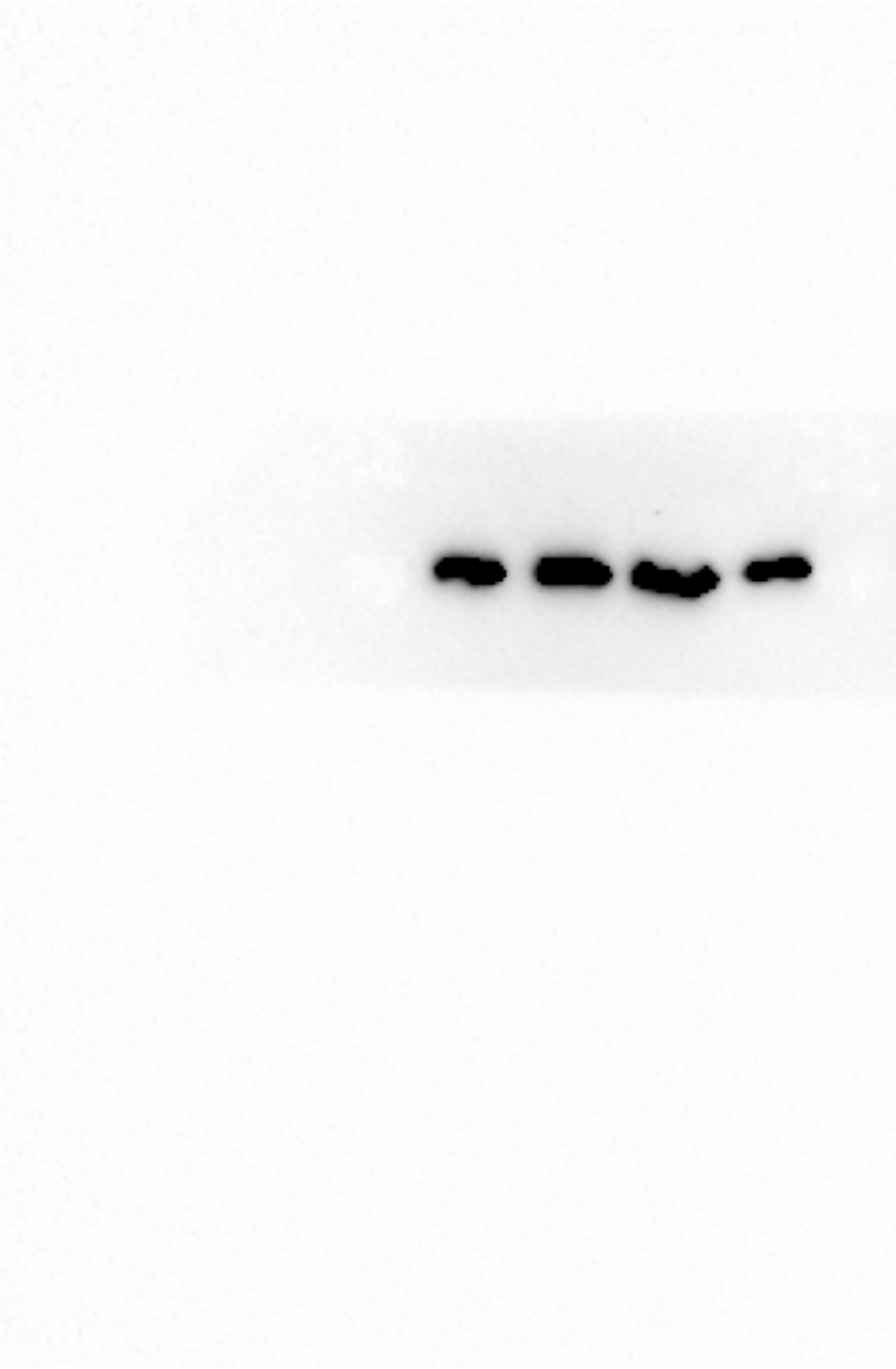

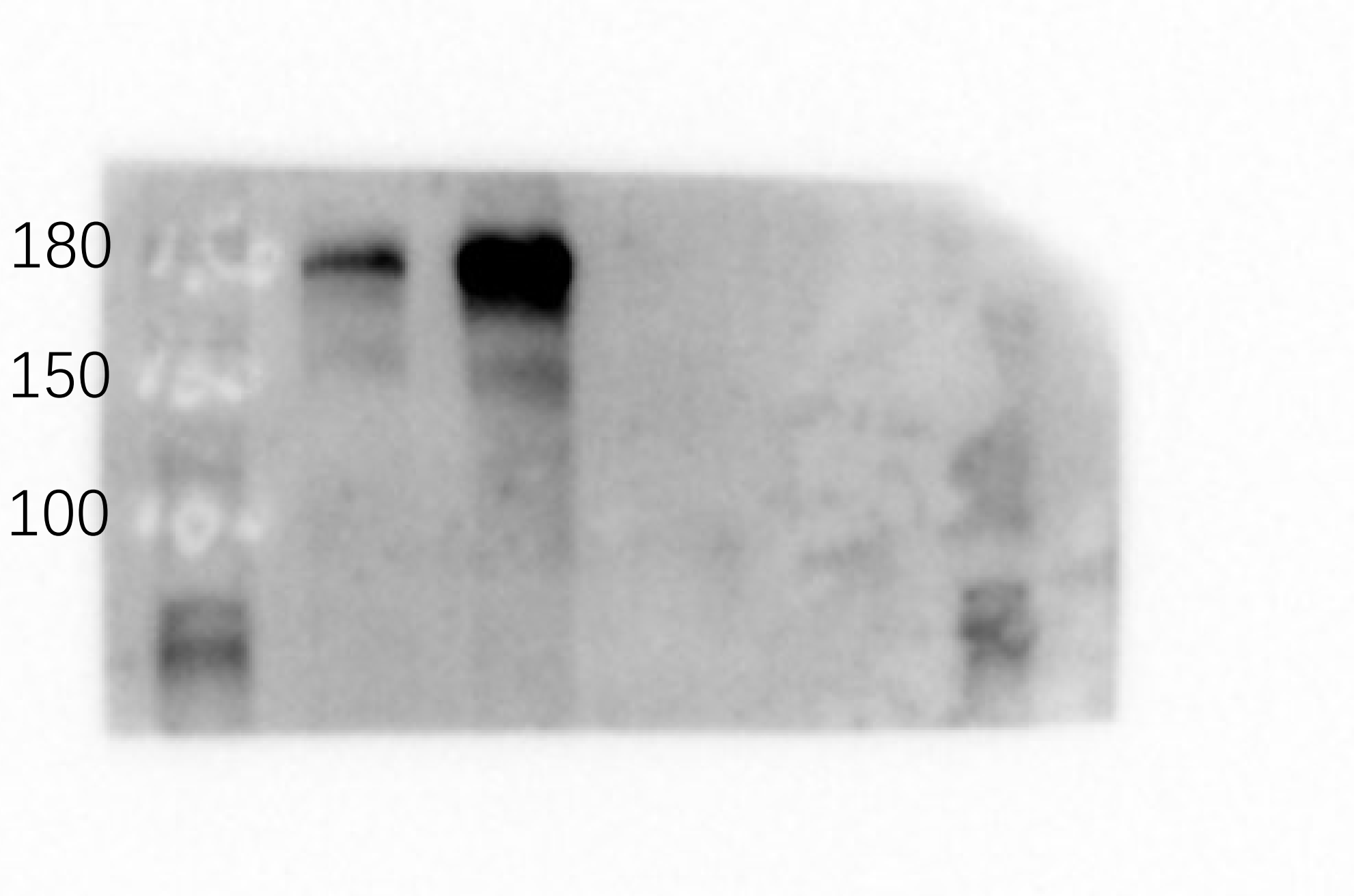
180
150
100

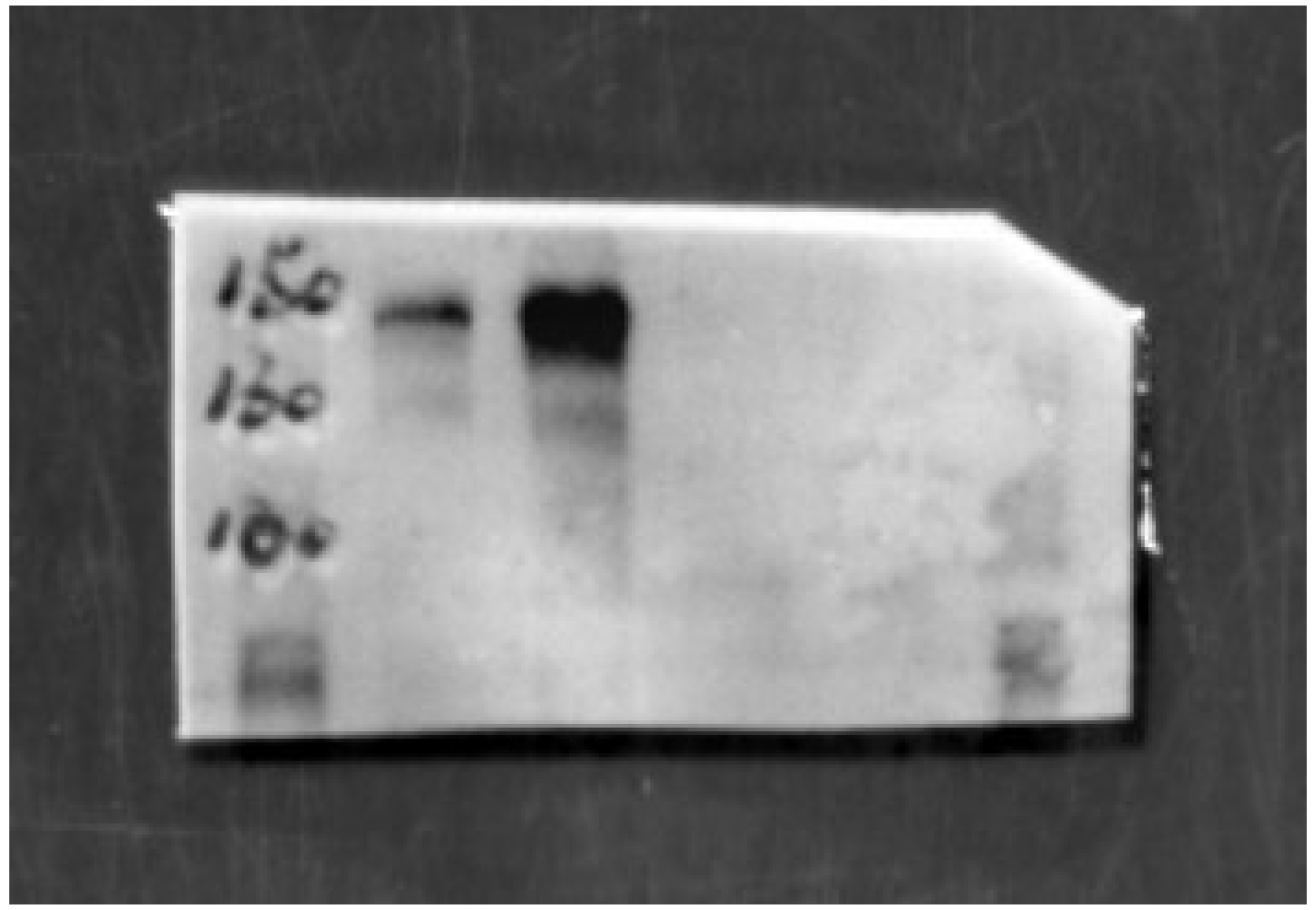

Supplement: Supplementary file 1 [file 41419_2026_8945_MOESM1_ESM.pdf]
