# Supplementary material for: Vascular SMC-like CAF-derived THBS1 drives tumor-associated neutrophil recruitment to orchestrate an immunosuppressive microenvironment in gastric cancer
Source: Cell Death Dis. 2026 Jun 3;17(1):694. doi: 10.1038/s41419-026-08945-1 (PMC13448681; doi:10.1038/s41419-026-08945-1)
Supplement: Supplementary file 2 [file 41419_2026_8945_MOESM2_ESM.docx]

**Supplementary Files**

**Supplementary Figure S1:** The proportion analysis of each cell type identified single-cell analysis of the GSE183904 dataset. (a) The proportion analysis of different cell types in per sample of the GSE183904 datasets. (b) The proportion analysis of each cell type in the GSE183904 cohort between normal samples and tumor samples. *, P < 0.05, **, P < 0.01

**Supplementary Figure S2:** The expression pattern of THBS1 in GC. (a) The IHC assays in GC tissues obtained from the Human Protein Atlas dataset were shown. THBS1 protein was expressed in vascular SMCs, SMCs, and Fibroblasts. (b) Analysis of the GSE13861, GSE26942, and GSE54129 GC cohorts showed that THBS1 expression was upregulated in GC. *, P < 0.05, **, P < 0.01

**Supplementary Figure S3:** Based on the Human Protein Atlas (HPA) dataset, we blasted the expression pattern of MMP9 in 18 immune cell types and total peripheral blood mononuclear cells (PBMC). The result showed that MMP9 was mainly expressed in neutrophils.

**Supplementary Figure S4:** THBS1 expression was positively correlated with tumor-associated macrophage infiltration level in the GSE122401 cohort.

**Supplementary Figure S5:** As the biomarker of vascular CAFs, MCAM was highly expressed in SMCs. The single-cell analysis of GC cohort was available in the DISCO platform.

**Supplementary Figure S1**

**Supplementary Figure S2**

MMP9 expression pattern

**Supplementary Figure S3**

**Supplementary Figure S4**

**Supplementary Figure S5**
